# A dataset of Agronomic Biofortification and Seeding rate - by - Location effects on Grain Mineral concentration, End-use quality and Agro-phenological traits of Durum wheat Genotypes

**DOI:** 10.1016/j.dib.2021.106899

**Published:** 2021-02-19

**Authors:** Anteneh Agezew Melash, Dejene K. Mengistu, Amare Assefa Bogale, Shegaw Getu Mengistu

**Affiliations:** a Debark University, College of Agriculture and Environmental Science, Department of Horticulture, P.O.Box, 90, North Gondar, Debark, Ethiopia; b Mekelle University, College of Dryland Agriculture and Natural Resource, Department of Dryland Crop and Horticultural Science, P.O.Box, 231; c Bioversity International, Ethiopia office, 1000 Addis Ababa, Ethiopia; d University of Gondar, College of Agriculture and Environmental Science, Department of Horticulture, Central Gondar, Ethiopia, P.O.Box, 196

**Keywords:** Data, Seeding rate, Micronutrients, Foliar application, Grain quality, Yield

## Abstract

Improving durum wheat end-use quality traits such as protein and gluten content becomes the principal research focus area, due to the increase in market demand and premium price paid for durum wheat producing farmers. The success is, however, limited because of crop genetic bottleneck, factors from growing environments and crop agronomic management practices. A study was conducted to i) identify an optimum seeding rate for durum wheat genotypes that can improve grain yield and grain quality and ii) to evaluate the effect of agronomic biofortification on grain protein, gluten contents, Zeleny index, and grain mineral content. Zinc and Iron containing fertilizers were applied foliarily in the form of ZnSO₄.7H_2_O and FeSO₄.7H_2_O. The data presented in this dataset article included yield and yield related traits, phenological and grain quality traits as well as grain Zn and Fe contents. The grain mineral content was measured by using atomic absorption photometer. A machine Minfra Smart T® wheat grain analyzer was used to measure grain protein content, gluten content, and Zeleny index values for each sample. The collected data were analyzed using GenStat (14th ed.) statistical software package. The aim of this dataset article is make the data publicly available to enable further extended analyses and as a guide for further research works to improve the productivity of smallholder durum wheat producing farmers.

## Specifications Table

**Table:** 

Subject area	Agricultural, Food and Nutrition Security
Specific subject area	Agronomy
Type of data	Table and Graphs
How data was Acquired	Various data types were collected by measurement in the field and laboratory. Some dataset were determined by using standard laboratory procedures.
Data format	Analyzed mean data and clean raw data
Parameters for data collection	Durum wheat genotypes were grown under a rain-fed condition with and without micronutrient fertilization. The test sites edaphic and climatic data were also collected. Mekelle site is characterized with neutral pH (6.9) and low soil organic matter (SOM) (1.2%) and total Nitrogen (ppm). The other site, Melfa, soil is clay loam with neutral pH (6.9) and very low in SOM (0.2%) content, total nitrogen (0.1%), and high in the soil available phosphorus (15.7 ppm). According to the soil atlas of the study areas, the soil of experimental sites are deficient in both Zn and Fe. The mean annual rainfall ranges between 412 – 762 mm. Two durum wheat genotypes, three levels of micronutrient, and four levels of seeding rate were considered and tested under contrasting environments. The grain samples were brought to the laboratory to detect grain protein, gluten, zinc, and iron contents.
Description of data collection	Phenological and agronomic data including days to 50% booting, days to 50% flowering, 90% maturity, plant height, seeds spike^−1^, spike length, tiller number, 1000-seed weight, biomass yield and grain yield were collected by measuring traits at the specified times in the experimental fields. Some traits were collected from five randomly sampled plants while others were collected on plot basis. Atomic Absorption Photometer was used to determine Zn and Fe concentrations in the grain samples. Minfra Smart® wheat grain Analyzer was used to measure grain protein content, gluten content and Zeleny index values for each sample from each genotype.
Data source location	Mekelle University (MU), northern Ethiopia, is the owner of the data. MU is located in Tigray region, at 13°30′N and 39°29′E with an elevation of 2210 meter above sea level. The second site “Melfa” is also located at 13°39′N and 39°10′E and an altitude of 2560 meter above sea level.
Data accessibility	With article and the raw data is deposited in the Mendeley dataset repository available at: https://data.mendeley.com/datasets/zpt52wg33c/1
Related research article	Anteneh A. Melash, Dejene K.Mengistu, Dereje A. Aberra, and Alemtsehay Tsegay (2019) The influence of seeding rate and micronutrients foliar application on grain yield and quality traits and micronutrients of durum wheat. Journal of Cereal Science 85, 221–227,225. https://doi.org/10.1016/j.jcs.2018.08.005

## Value of the Data

• This dataset provides valuable information on foliar based application of micronutrient as agronomic management to improve concentrations of Zn and Fe micronutrients in durum wheat grain. Such agronomic biofortification plays great role in reducing the acute health problem related to deficiency of micronutrients in developing countries. This influences policy makers and research system to focus on breeding for micronutrient accumulation and agronomic biofortification rather than depending on expensive artificial grain and flower fortification approaches to overcome the defficiency of micronutrients. This information can help to link agriculture with the health sector, as malnutrition and associated problems are partially associated with deficiency of micronutrients in the human body.
• The dataset in this article provides information about defining and optimization of seeding rate under dryland conditions of northern Ethiopia and countries that share similar agro-ecology. The dataset further illustrates the factors that affect the determination of the optimum seeding rate for a specific location.
• Data can be used further in breeding programs of durum wheat, especially to fortify the grain micronutrient concentration through genetic manipulation.

## 1 Data Description

Major pre-data collection activities including variety selection, change of seeding rates, application of micronutrients, and selection of suitable growing locations are key elements in the bio-fortification program and improvement of grain quality traits. This dataset article describes the factors that affect the agronomic traits, phenological traits, grain micronutrient content, and grain quality traits of durum wheat (Tables 1–5). The data presented in Table 1 showed the interaction effect between durum whaet genotypes and seeding rate on agronomic and phenological traits of durum wheat. Table 2 provides an important interaction effect between seeding rate and growing location to influence some agronomic traits of durum wheat genotypes. Variations in grain yield and yield components across tested genotypes and seeding rates gave interesting insights to consider a selection of durum wheat genotypes and seeding rate effects for their improvement (as shown in Table 3). Table 4 contains grain yield and yield components as influenced by adjustment of seeding rate, variation in growing location, and varietal difference. Table 5 preseted the interaction effect of genotype, foliar micronutrient application, and adjustment of seeding rates on some agronomic measurements, above-ground biomass, and grain yield. This dataset article provides the raw data associated with each and individual repeat in all experimental conditions and thus, the raw data is deposited in the Mendeley dataset library.

**Table 1:** Mean values for the combined effects of durum genotypes and adjusted seeding rates on some selected phenological traits.

		Selected phenological traits		
Genotypes	Seeding rate (kg ha^−1^)	50% DB	50% DF	DM
208,304	**100**	62.1	68.6	99.5
	**125**	62.8	68.6	101.6
	**150**	61.8	68.6	100.8
	**175**	61.3	68.6	102.1
Asassa	**100**	58.8	66.3	100.5
	**125**	60.4	67.7	101.0
	**150**	59.6	66.8	100.6
	**175**	59.7	66.9	100.8

LSD _0.05_		1.9	1.2	2.0
CV (%)		0.9	1.4	1.0

**Table 2:** Mean values for the combined effects of adjustment in seeding rates and growing location on some selected agronomic measurements of durum wheat.

		Agronomic measurements						
Location	Seeding rate (kg ha^−1^)	PH (cm)	NET	SPL (cm)	SPS	BY (t ha^1^)	GY (t ha^−1^)	TGW (g)
Melifa	**100**	92.2	4.5	6.2	43.5	7.6	2.1	39.7
	**125**	91.7	3.6	6.0	39.1	6.8	2.1	38.6
	**150**	93.7	3.8	6.0	40.2	7.3	2.5	37.1
	**175**	91.5	3.5	5.6	39.4	7.1	2.2	35.4
MU	**100**	78.8	3.6	5.1	34.4	4.9	1.3	33.4
	**125**	82.4	4.0	5.2	34.6	6.3	1.7	35.4
	**150**	79.7	3.8	5.1	33.1	6.4	1.6	32.4
	**175**	82.6	3.9	5.0	34.4	7.9	1.9	32.7

LSD _0.05_		1.4	0.5	0.4	2.1	0.7	0.1	0.8
CV (%)		2.0	13.9	9.3	6.7	9.5	4.9	2.8

**Keys to abbreviations; *SPL:****spike length,****NET:****number of effective tiller,****SPS:****seeds spike^−1^,****BY:****biomass yield,****GY:****grain yield,****TGW:****thousand grain weight,***PH*:****plant height,****CV:****coefficient of variation,***LSD_0.05_:***least significant difference at 5% level of probability.*

**Table 3:** Mean values for the combined effects of seeding rates and foliar based micro nutrient application on some selected agronomic measurements and grain yield of durum wheat.

Micro nutrients	Seeding rate (kg ha^−1^)	PH (cm)	NET	SPL (cm)	SPS	BY (t ha^−1^)	GY (t ha^−1^)	TGW (g)
Control	**100**	87.0	4.8	5.7	39.2	6.3.	1.6	35.2
	**125**	87.0	4.1	5.8	36.4	6.5	1.7	35.0
	**150**	87.8	3.9	5.7	37.7	6.8	2.0	34.0
	**175**	91.4	3.9	5.3	37.8	7.9	2.1	33.4
FeSO_4_	**100**	86.0	3.5	5.6	39.4	6.7	1.9	37.1
	**125**	85.5	3.1	5.5	36.0	5.6	1.8	38.7
	**150**	85.5	3.9	5.5	35.7	6.8	1.9	35.1
	**175**	82.2	3.6	5.1	35.2	7.2	1.8	34.1
ZnSO_4_	**100**	83.6	3.7	5.6	38.3	5.8	1.6	37.4
	**125**	88.6	4.2	5.6	38.1	7.6	2.1	37.2
	**150**	86.8	3.6	5.4	36.7	6.8	2.1	35.1
	**175**	87.6	3.5	5.5	37.6	7.4	2.2	34.6

LSD _0.05_		1.6	0.8	0.5	2.4	0.9	0.1	1.2
CV (%)		1.3	10.5	4.1	4.7	9.5	5.0	1.7

**Table 4:** Summary of significance among genotype, seeding rates, and location interactions on yield and growth measurements of durum wheat genotypes.

		PH (cm)		NET		SPL (cm)		SPS		BY(t ha^−1^)		GY(t ha^−1^)		TGW(g)	

Genotypes	Seeding Rate (kg ha^−1^)	Melifa	MU	Melifa	MU	Melifa	MU	Melifa	MU	Melifa	MU	Melifa	MU	Melifa	MU
208,304	**100**	93.3	82.9	4.1	6.0	5.1	3.8	36.9	31.0	6.9	5.4	2.2	1.3	35.2	34.2
	**125**	92.5	85.0	3.1	6.0	4.9	3.7	32.1	29.4	7.3	6.1	2.3	1.7	35.8	37.7
	**150**	94.7	83.1	3.8	5.9	5.1	3.8	37.0	29.7	8.2	6.2	2.5	1.4	34.4	32.0
	**175**	92.5	84.3	3.8	5.6	4.9	3.6	33.2	30.6	7.5	7.6	2.3	1.7	31.5	34.4
Asassa	**100**	91.1	74.7	4.9	6.3	5.1	3.5	50.1	37.8	8.3	4.4	1.9	1.4	44.2	32.7
	**125**	90.9	79.8	4.0	6.1	5.4	4.3	46.1	39.8	6.3	6.7	2.0	1.7	41.4	33.1
	**150**	92.6	76.3	3.9	6.0	5.2	3.7	43.4	36.6	6.5	6.3	2.4	1.7	39.9	32.7
	**175**	90.4	80.9	3.1	5.7	5.1	4.1	45.5	38.1	6.7	8.2	2.2	2.2	39.3	31.1

LSD_0.05_		1.9		0.6		0.9		2.8		1.2		0.1		1.0	
CV (%)		1.3		10.5		4.1		4.7		11.7		5.0		1.7

**Table 5:** Summary of significance *(P < .05)* for genotype, seeding rates and micro nutrients interaction on yield and yield attributed measurements of durum wheat under two contrasting growing locations.

			NET		SPL		SPS		BY (t ha^−1^)		GY (t ha^−1^)		TGW (g)	

Genotypes	Micro nutrients	Seeding rate (kg ha^−1^)	Melifa	MU	Melifa	MU	Melifa	MU	Melifa	MU	Melifa	MU	Melifa	MU
208,304	**Control**	**100**	4.5	4.6	6.1	5.2	41.3	34.4	6.8	6.5	2.0	1.1	35.8	31.5
		**125**	3.4	3.2	6.1	5.0	32.8	24.5	6.8	5.2	2.2	1.0	37.9	29.4
		**150**	4.8	3.8	6.3	5.2	40.0	27.6	5.7	6.0	1.9	1.2	35.8	23.1
		**175**	4.2	3.6	5.6	4.7	36.0	34.5	6.3	8.9	2.0	2.0	31.5	31.5
	**FeSO_4_**	**100**	3.6	3.3	5.9	5.1	32.1	30.2	7.8	6.3	2.3	1.8	33.7	37.5
		**125**	2.2	3.1	6.0	4.7	31.2	30.1	7.3	4.2	2.4	1.4	33.7	41.9
		**150**	3.7	3.6	5.7	5.0	37.0	29.0	9.9	5.4	3.2	1.1	33.7	39.7
		**175**	3.5	3.4	5.3	4.7	29.8	26.2	8.3	5.5	2.3	0.9	29.4	35.8
	**ZnSO_4_**	**100**	4.3	3.5	6.1	5.0	37.3	28.4	6.3	3.4	2.1	0.9	36.3	33.7
		**125**	3.7	4.9	5.8	5.0	32.2	33.7	7.8	8.9	2.4	2.5	35.8	41.6
		**150**	2.8	4.0	5.6	5.0	34.1	32.4	8.9	7.3	2.4	2.1	33.7	33.3
		**175**	3.6	3.9	5.8	5.3	33.9	31.0	7.8	8.3	2.5	2.2	33.7	35.8
Asassa	**Control**	**100**	6.3	3.8	6.6	5.1	46.0	35.1	7.3	4.7	2.1	1.2	44.2	29.3
		**125**	4.4	5.3	6.0	5.6	43.4	44.8	6.8	7.3	1.6	2.1	42.0	30.6
		**150**	3.0	4.1	5.7	5.4	42.0	41.1	7.3	8.3	2.3	2.7	39.8	37.2
		**175**	2.8	5.1	6.1	4.9	40.4	40.1	6.0	10.4	1.9	2.8	37.9	32.7
	**FeSO_4_**	**100**	4.3	3.5	6.2	5.2	55.1	40.0	7.3	5.5	1.5	2.0	42.0	35.2
		**125**	3.2	3.8	5.9	5.6	44.7	38.1	4.2	6.8	1.8	1.8	42.0	37.3
		**150**	4.7	3.5	6.3	5.1	44.8	31.8	5.5	6.3	2.2	1.3	37.9	29.3
		**175**	3.6	3.8	5.6	5.0	49.6	35.2	7.8	7.0	2.5	1.6	40.0	31.4
	**ZnSO_4_**	**100**	4.0	3.1	6.1	5.1	49.1	38.2	10.4	3.1	2.2	1.1	46.3	33.5
		**125**	4.5	3.7	6.4	5.6	50.1	36.5	7.8	6.0	2.5	1.3	40.0	31.4
		**150**	4.0	3.5	6.1	5.0	43.5	36.9	6.8	4.4	2.8	1.2	42.0	31.5
		**175**	3.0	3.5	5.4	5.4	46.4	39.1	6.3	7.0	2.0	2.1	40.0	29.0

LSD _0.05_			**1.2**		**1.1**		**4.8**		**1.8**		**0.2**		**2.0**	
CV (%)			**13.9**		**9.3**		**6.7**		**11.2**		**4.9**		**2.8**	

## 2 Experimental Design, Materials and Methods

Replicated studies were carried out at two divergent growing locations at Mekelle University research station and (13°30′N and 39°29′E) and Melfa farmers training center (13°39′N and 39°10′E). These locations have a warm and moist climate, representative of northern Ethiopia. The experimental treatments included four seeding rates (i.e. 100 kg ha^−1^, 125 kg ha^−1^, 150 kg ha^−1^, and 175 kg ha^−1^), three micronutrients, and two durum wheat genotypes, *Asassa* and *Rigeat*. Both varieties where characterized for their terminal drought tolerance nature. The treatments were arranged in a split-split plot design, with two replications. The genotypes were assigned into the main plots, seeding rates into the sub-plots and micronutrients into the sub-sub-plots. ZnSO₄.7H_2_O and FeSO₄.7H_2_O were used as a source of zinc and iron, respectively. It was applied foliar at the early grain filling stage of the crop, both at a rate of 25 kg ha^−1^. This rate was hypothetical sufficient for the study areas based on the soil atlas of the experimental locations [1]. The net plot size of sub-plots and sub-sub-plots was 4.4 *m* × 2.5 m and 1.2 *m* × 2.5 m, respectively. Each sub-plot and sub-sub-plots were separated by 0.5 m and 0.4 m spacing, respectively. The spacing between replications was also 0.8 m spacing. All experimental plots were equally treated with 46 kg ha^−1^ nitrogen and 20 kg ha^−1^ of phosphorus. The nitrogen was split into two does where the first half-dose together phosphorus was applied at planting. The remaining half-dose of nitrogen was applied at the tillering stage. Weed control was done manually and maintained at bay throughout the cropping season.

### 2.1 Data collection

#### 2.1.1 Agronomic traits

Agronomic traits were collected at both experimental locations. Five representative plants from each experimental plots were randomly selected from the central four rows to measure seeds spike^−1^, spike length (cm), plant height (cm), and tiller number. A digital scale was used to measure the above ground-biomass and measuring tape was used to measure the spike length and plant hight.

#### 2.1.2 Grain mineral concentration analysis

Durum wheat grains were ground into fine flours to detect grain zinc and iron concentration. The analysis was performed at EZANA mining development PLC laboratory, Ethiopia. Twenty grams of each sample was weighed and digested using Perten Laboratory Mill 120 to 0.8 mm standard sieve. The grain Zn and Fe content were determined using Varian AA240FS Fast Sequential Atomic Absorption Photometer, operated with SpectrAA base and PRO software was used to analyze Zn and Fe content in the grain. All required grain samples from each genotype and location were digested in an automated digestion chamber. The grain Zn and Fe concentration (mg kg^−1^) was measured as described by Kunda et al., [2].

#### 2.1.3 Grain quality traits measurements

Three hundred and fifty grams of harvested whole-grain subsamples from each plot were packaged and sent to Sinana Agricultural Research Center (SiARC) wheat laboratory for analysis for grain protein content, gluten content and Zeleny index using Minfra Smart® wheat grain Analyzer.

#### 2.1.4 Grain yield and 1000-grain weight

Grain yield of each plot was collected by harvesting and threshing the four central rows. Thousand grain weight was obtaind by counting 1000 grains from the harevest. both Grain yield and 1000 grain weight were adjusted to 12.5% grain moisture content as described by Badu-Apraku et al. [3].

$$\text{Grain}\;\text{yield}\;\left(\text{kg}\;ha^{-1}\right)=\frac{100\;-\;\%\;\text{AMC}}{100\;-\;\%\;\text{SMC}}\times 100$$

**Where,**

**AMC** is the percent of actual grain moisture content (%) and **SMC** is the percent of standard grain moisture content

#### 2.1.5 Phenological traits

Phenological traits such as 50% days to booting, 50% days to flowering, and 90% of days to physiological maturity were recorded from both experimental sites. The stages were defined and recorded following Zadok's wheat growth code. The 50% booting date was recorded for about 50 percent of the crop plot^−1^ starts to opening the flag leaf sheath (GS 47). Similarly, the data for 50% flowering was recorded when 50% of the spike per plot have shown the anther (GS 65) [4]. We also recorded the days to maturity when the peduncles turned into golden yellow color.

### 2.2 Statistical data analysis

The raw data, after checking for normality and homogeneity, was analyzed for variance using GenStat statistical software ver. 14th [5], following a split – split-plot design structure. This analysis allowed us to examine the main effects and combined effect of durum wheat genotypes, the adjusted seeding rates, and foliarly applied micronutrients. For significant effects, means were separated using the least significance difference (LSD) at a 5% significance level. Duncan's Multiple Range Test (DMRT) was employed for comparison of the various interaction means presented in various graphs. The mean values were used to construct the graphs using excel graphing features. Bars representing treatment combination means were designated by error bars and separated by letters where bars separated by different letters were significantly different from each other.

## CRediT Author Statement

**Mr. Anteneh Agezew Melash:** has conceptualized the research, develop the method, laid out the experiment, collect the data, writing - the original draft, investigation, and makes a formal analysis; **Dr. Dejene Kassahun Mengistu:** (an associate professor of plant genetic resources at Mekelle University) supervise the research project, validate, investigate, make a formal analysis, reviewing and editing the research papers; **Mr. Amare Aseffa** and **Mr. Shegaw Getu:** were reviewed and edited the research article as well.

## Declaration of Competing Interest

The authors declare there is no known competing interest.

## Data Availability

A raw data relative to each experimental repeat of Agronomic traits, Grain quality traits and Grain micronutrient content as influenced by seeding rate, variety and micronutrients (Original data) (Mendeley Data).

## References

[1] ATA, *(Agricultural Transformation Agency)* (2014). Soil Fertility Status and FertilizerRecommendation Atlas for Tigray regional state, Ethiopia. Ministry of agricultureand Ethiopia Agricultural Transformation Agency.

[2] Badebo A., Gelalcha S., Ammar K., Nachit M., Abdalla O. (2009). Overview of Durumwheat Research in Ethiopia: Challenges and Prospects. https://www.cabdirect.org/cabdirect/abstract/20103156334.

[3] Kundua S., Poddera R., Betta K., Schoenaub K., Vandenberg A. (2017). Optimizing seed sample size for zinc and iron analysis of wild and cultivated lentil. J. Commun. Soil Sci. Plant Anal..

[4] Zadoks J., Chang T., Konzak C. (1974). A decimal code for the growth stage of cereals. Weed Res..

[5] Payne R., Murray D., Harding S., Baird D., Soutar D. (2011). An Introduction to GenStat for Windows.

